# Effects of Stevia Straw Supplementation on Meat Quality, Nutrient Composition, and Rumen Microbiota in Sheep

**DOI:** 10.3390/vetsci12101018

**Published:** 2025-10-21

**Authors:** Congbin Xu, Yan Ma, Jinlong Li, Tuo Yong, Liangzhong Hou, Tongjun Guo

**Affiliations:** 1 Institute of Feed Research, Xinjiang Academy of Animal Science, Urumqi 830011, China; 2 Xinjiang Key Laboratory of Herbivorous Livestock Feed Biotechnology, Urumqi 830011, China; 3 College of Animal Science, Xinjiang Agricultural University, Urumqi 830011, China

**Keywords:** *stevia* straw, Bayinbruk sheep, microbiota, meat quality fatty acids

## Abstract

This study investigated the effects of dietary *stevia* straw supplementation on meat quality, amino acid and fatty acid content, and rumen microbial diversity in sheep. A total of 50 ram lambs (3–4 months, 27.01 ± 3.8 kg) were randomly assigned to five groups. Each group received a total mixed ration supplemented with 0% (control), 5%, 15%, 25% and 35% dry *stevia* straw for 72 days. Adding 15% and 25% *stevia* straw significantly increased the pre-slaughter body weight. EE content was also significantly higher across all experimental groups (*p* < 0.01), showing a linear increase with supplementation. Glutamic acid content in the 15% group was significantly higher than that in the 25% group (*p* < 0.05). The addition of stevia straw improved the fatty acid profile: C18:1n9c content in the 5%, 15%, and 25% groups was significantly higher than that in the CK (Control) group (*p* < 0.05); C18:2n6c content in the 25% group was significantly higher than that in the CK, 5%, and 15% groups (*p* < 0.05); the content of (C20:1) was extremely significantly higher in all experimental groups compared to the CK group. Additionally, the unsaturated fatty acid content in the 25% group was significantly higher than that in the CK group (*p* < 0.05). The relative abundances of Bacteroidota and Firmicutes were increased in the 25% and 35% groups, but the differences were not significant (*p* > 0.05). In conclusion, dietary supplementation of 15–25% stevia straw to the ration can significantly improve the slaughtering performance, meat quality and muscle nutrient composition of fattening sheep. In addition, the addition of stevia straw had no negative effect on the rumen microflora, and the 15% level could increase the abundance and activity of the rumen microflora, so it is recommended to use 15% stevia straw in the ration for sheep during the fattening period.

## Simple Summary

*Stevia* (*Stevia* rebaudiana), commonly referred to as sweet grass or honey leaf, is an annual herbaceous plant of the *Stevia* genus in the Compositae family. *Stevia* straw, composed of stems and roots remaining after leaf harvesting, is highly nutritious and rich in various bioactive compounds. It has been shown to regulate livestock production performance, improve the quality of animal products, and enhance economic benefits in animal farming. This study demonstrates that dietary supplementation of 15–25% *stevia* straw to the ration can significantly improve the slaughtering performance, meat quality and muscle nutrient composition of fattening sheep. In addition, the addition of *stevia* straw had no negative effect on the rumen microflora, and the 15% level could increase the abundance and activity of the rumen microflora, so it is recommended to use 15% *stevia* straw in the ration for sheep during the fattening period.

## 1. Introduction

As China’s economic development continues to improve, people’s demand for meat, eggs, milk and other livestock products has also risen. However, the rapid development of animal husbandry has made the imbalance between forage supply and demand increasingly prominent. Due to the limited area of arable land, it is a challenge to take into account the cultivation of forage crops while meeting the demand for human food, thus triggering the contradiction of “people and animals competing for food”, which not only restricts the sustainable development of animal husbandry, but also further exacerbates the shortage of forage; therefore, the development of new forage resources has become a major goal.

*Stevia* is an annual herbaceous plant of the Stevia genus in the Compositae family [1], and its leaves are rich in a variety of active substances widely used in medicines and food [2]. In China, *stevia* is mainly grown in Xinjiang, Jiangsu, Anhui and other regions, and the cultivation area reached 300,000 mu (49,421 acres) in 2023, with an annual output of dry *stevia* leaves of 75,200 tons and *stevia* straw of 106,000 tons. *Stevia* straw is the by-product remaining after leaf harvesting, and its utilization could alleviate the shortage of forage for cattle and sheep raising, and the nutrient levels were better than those of conventional straw roughage [3]. The Bayanbulak sheep, also known as the Tianshan Yak Sheep or Bayanbulak Large-Tailed Sheep, is a unique breed primarily distributed in the Hejing County of the Bayingolin Mongol Autonomous Prefecture in Xinjiang Uygur Autonomous Region, China, specifically in the Bayanbulak Grassland. It belongs to the meat-and-fat dual-purpose coarse-wool sheep category and is renowned for its exceptional environmental adaptability, superior meat quality, and distinctive physical characteristics. Utilizing stevia straw as sheep feed is an innovative strategy that kills multiple birds with one stone. Economically, it creates new industrial chains and optimizes resource allocation. In terms of profitability, it provides farms with a powerful tool to reduce costs and enhance efficiency, provided that technical and managerial challenges are addressed. Environmentally, it represents a typical low-carbon, circular development path with significant ecological benefits.

In addition, *stevia* straw contains bioactive compounds such as glycosides, chlorogenic acids and flavonoids, which have a positive effect on the growth and immune function of livestock and poultry and can be used as feed additives to promote their healthy growth [4]. Shin et al. [5] observed that dietary supplementation with stevioside at 1625 mg/kg BW significantly increased the average daily gain and reduced the drip loss and shear force of the longissimus dorsi muscle in beef cattle. Zhang et al. [3] found that the addition of 1.0% *stevia* straw to cattle feed increased feed conversion and promoted rumen fermentation. Adding 4% and 6% *stevia* residue to laying hens’ diets increased the relative abundance of beneficial bacteria in the intestinal tract, decreased the number of harmful bacteria, and improved the amino acid and fatty acid compositions of the egg whites [6]. Currently, a substantial amount of *stevia* straw is wasted due to difficulties in harvesting and low utilization efficiency in feed processing. However, *stevia* straw possesses considerable potential for animal feeding and medicinal applications. Therefore, this study aimed to evaluate how including varying levels of *stevia* straw in sheep diets affects slaughter traits, meat quality, amino acid and fatty acid profiles, rumen fermentation parameters, and microbial diversity, thereby providing an empirical foundation for its use in ovine production.

## 2. Materials and Methods

### 2.1. Experimental Materials

**Figure 1:**
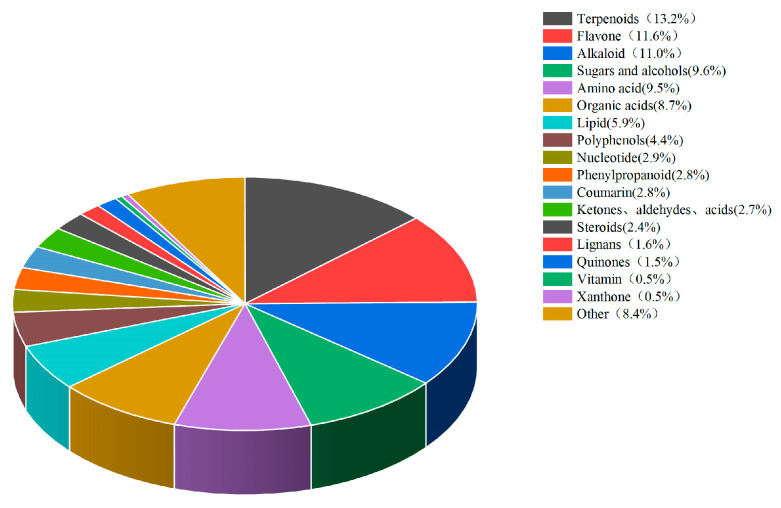
Classification of bioactive substances in *stevia* straw.

Stevia straw was provided by the Xinjiang Shuomu Breeding Co. Ltd. in Heshuo County, Bayin’guoleng Mongol Autonomous Prefecture, Xinjiang, and was the residual product (mainly stems) of *stevia* after leaf harvest. The nutrient composition of *stevia* straw is shown in Table 1. Samples of *stevia* straw were sent to Beijing Baimaike Biological Co. Ltd. for the quantification of widely targeted metabolites using liquid chromatography-mass spectrometry (LC-MS). A total of 1183 biologically active substances were detected, which were classified into 18 types, with terpenoids accounting for the highest proportion (13.2%), followed by flavonoids (11.6%), alkaloids (11.0%), sugars and alcohols (9.6%) in Figure 1.

### 2.2. Animal Handling, Diets, and Experimental Design

All animal procedures in this study were approved by the review board of the Protocol Management and Review Committee of the Feed Research Institute of the Xinjiang Animal Science Academy (No. 11 20231227).

The study was designed as a completely randomized one-way trial. Fifty male Bayinbruk lambs, 3–4 months of age and weighing 27.01 ± 3.8 kg were randomly grouped based on their initial weight, and were divided into five groups, with ten sheep in each group. An isoenergetic and isonitrogenous diet during the fattening period, and with a standard nutritional requirement for a 250 g daily gain, sheep weighing 30 kg were selected to receive a mixed pelleted ration containing 0% (CK), 5%, 15%, 25% and 35% *stevia* straw; the specific compositions and nutrient levels of the various groups are shown in Table 2.

Prior to the formal trial period, the enclosures were disinfected, and the sheep were dewormed. Five of these groups were raised in five different enclosures. The sheep were then reared in groups in the same pen. They were fed ad libitum (twice a day at 9:00 and 19:00) and had free access to water. The total experimental period lasted 82 days, including a 10-day pre-feeding period and 72-day trial period.

### 2.3. Sample Collection

Upon completion of the trial, following 12 h of fasting and 2 h of water restriction, five lambs from each group were randomly selected and transported to the slaughterhouse. After humane slaughter, the backfat thickness was measured vertically to the skin surface at the junction of the 12th and 13th ribs, 5 cm from the dorsal midline, using a vernier caliper with an accuracy of 0.1 mm. Upon completion, the longissimus dorsi muscle was collected from between the 12th and 13th ribs and preserved in liquid nitrogen. Rumen fluid samples were collected: the rumen was removed after slaughter, and the rumen fluid was collected from the upper opening of the left side. In order to avoid contamination of the rumen fluid, the first 50 mL of rumen fluid was discarded, and 30 mL of rumen fluid was collected after 4 layers of gauze filtration, and the rumen pH was measured immediately using a portable pH meter ((Shanghai Yidian Scientific Instrument Co., Ltd., Shanghai, China), Shanghai, China). A total of 10 mL of rumen fluid was transferred to −20 °C and stored in the freezer. for subsequent detection and analysis. Another 5 mL of rumen fluid was collected into a freezing tube and stored at −80 °C after liquid nitrogen flash-freezing for the determination of rumen microbiota.

### 2.4. Measurement of Indicators

#### 2.4.1. Carcass Traits

The live weight (kg) of each experimental sheep was measured and recorded just before slaughter. The test sheep were bled through the carotid artery and jugular vein, and the head, hoof, skin, and viscera of the test sheep were removed, while the kidneys were retained. The carcass weight was measured and recorded immediately after slaughter, and slaughter percentage was calculated as carcass yield = (carcass weight/live weight) × 100%. A longitudinal incision was made on the longissimus dorsi (LD) muscle between the 12th and 13th ribs to obtain the area of the eye muscle. A Vernier caliper was used to measure the length of the longest and widest parts of the eye muscle. The eye muscle area is the product of the length and width of the eye muscle multiplied by 0.7.

#### 2.4.2. Meat Quality

Meat quality was assessed through both physical and chemical indicators. Physical measurements comprised pH, water loss (%), and cooking loss (%), while chemical analyses included moisture, protein, fat, and ash content.

Muscle pH was measured using a portable muscle tissue pH meter (PHS-3E; Shanghai YiDian Scientific Instrument Co., Ltd., Shanghai, China), calibrated using standard buffer solutions at pH = 4, pH = 6.86, and pH = 9.18. To calculate water loss (%), longissimus dorsi muscle samples were cut perpendicular to the muscle fiber direction into 1 cm thick slices. A circular sampler with a diameter of 3 cm (DL-100; Sunspring Sanquan Zhongshi, Shandong, China) was used to obtain samples. The samples were placed between six layers of filter paper and pressed under 35 kg of pressure for 5 min. The weights before and after pressing were measured, and the water loss rate was calculated using the following formula: water loss rate (%) = (Weight before pressing − Weight after pressing)/Weight before pressing × 100. Cooking loss was evaluated by drying a 3 × 3 × 5 cm meat sample with a paper towel, recording its initial weight (W1, g), sealing it in a bag, and heating it in an 85 °C water bath for 40 min. After cooling and drying, the sample was reweighed (W2, g); cooking loss (%) = (W1 − W2)/W1 × 100. Moisture, crude protein, crude fat, and ash contents were quantified following standard AOAC methods [7]: 950.46, 928.08, 960.39, and 920.153, respectively.

#### 2.4.3. Cholesterol, Inosinic Acid, Thiamine

Weigh about 2 g of sample in a 50 mL centrifuge tube, homogenize with 20 mL of 5% perchloric acid solution three times, and centrifuge the homogenate at 4000 r/min for l0 min at 4 °C. Then, transfer the supernatant into a 50 mL beaker, and continue to wash the precipitate with 5% perchloric acid and centrifuge it two times, merge the supernatant, adjust the pH with KOH solution to 6.5, and shake the supernatant well with distilled water until it is 50 mL. Then, filter it through a 0.45 μm filter membrane and put it into a bottle for on-line testing. Filter with a 0.45 μm membrane and put into a bottle for testing. Chromatographic column: C18 (length 250 mm, inner diameter 4.6 mm, particle size 5 μm); column temperature: 25 °C; injection volume: 20 μL; flow rate: l mL/min; detection wavelength: 254 nm; mobile phase: take 3.5 mL of phosphoric acid solution and add 200 mL of water and 7.2 mL of triethylamine mixing, and then adjust the pH of the solution to 6.5 with triethylamine, and then take 950 mL and 50 mL of methanol mixing. A total of 50 mL of methanol was mixed, filtered through 0.45 μm and degassed by ultrasonic.

#### 2.4.4. Amino Acids

To determine amino acid profiles, 100 mg of lyophilized tissue was homogenized in 1.2 mL of 10% sulfosalicylic acid and centrifuged at 13,500× *g* for 15 min at 4 °C. The supernatant was collected, filtered through a 0.22 μm membrane, transferred to a 2.0 mL glass vial, and analyzed using a high-speed amino acid analyzer (L-8900; Hitachi High-Tech Corporation, Tokyo, Japan).

#### 2.4.5. Fatty Acids

Total fatty acids (FAs) were extracted from frozen meat samples following the procedure of Liang et al. [8]. FA separation was performed using gas chromatography (GC-450; Varian Co., Walnut Creek, CA, USA), with peaks identified based on retention time. Individual FA concentrations were quantified against standard curves prepared from a known methyl ester mixture (C4–C24; Sigma-Aldrich, St. Louis, MO, USA)

#### 2.4.6. Rumen Fluid Sample Collection and Analysis

DNA was purified from rumen fluid by the cetyltrimethylammonium bromide (CTAB) method and used as template for PCR amplification with specific primers [8]. The PCR products were quantified by fluorometry (Qubit 3.0), and sequenced on an Illumina PE300 platform at the Beijing Baimaike Biological Co. The raw data from 16S rRNA gene sequencing was QC processed with QIIME2, to remove low-quality reads and potential contaminants. The SILVA database was selected for alignment, and a 97% sequence similarity cut-off was used to identify operational taxonomic units (OTUs). Alpha diversity of the microbial community was assessed by Chao1, ACE and Shannon indexes in QIIME2. Differences in relative bacterial abundance were evaluated by the nonparametric Kruskal–Wallis rank-sum test, and the difference between groups was analyzed by Student’s *t*-test. Bacterial markers differentiating the various microbial communities were determined by linear discriminant analysis (LDA) effect size (LEfSe) (LDA > 3.5, *p* < 0.05).

### 2.5. Statistical Analysis of Data

Data are expressed as means ± standard errors of the mean. Data were preliminarily sorted using Excel2010, and SPSS24.0 was used for performing one-way ANOVA. If the difference between treatments was significant, a Duncan multiple-range test was used for comparisons, and orthogonal polynomials were used to evaluate the linear and secondary effects of changes in relevant data. We used 0.05 ≤ *p* < 0.10, *p* < 0.05, and *p* < 0.01 to indicate a significant trend, significant difference, and highly significant differences, respectively.

## 3. Results and Analysis

### 3.1. Carcass Traits

As shown in Table 3, the live weight before slaughter showed a quadratic change with increasing *stevia* straw addition, first rising and then falling (*p* = 0.02). The 15% and 25% groups were significantly higher than the CK group, with an increase of 24.86% and 15.91%, respectively (*p* < 0.05). The carcass weight showed a quadratic change with increasing addition of *stevia* straw, initially increasing and then decreasing (*p* = 0.02). The other indicators were either rising or falling compared with the CK group, but there was no significant difference (*p* > 0.05).

### 3.2. Effect of Different Levels of Stevia Straw on Lamb Meat Quality

As shown in Table 4, the rate of water loss followed a quadratic curve of decreasing and then increasing with an increasing addition of *stevia* straw (*p* < 0.01); the 15% and 25% groups were significantly lower than the CK group, with a reduction of 6.93% and 7.44%, respectively (*p* < 0.05). The cooking loss likewise showed a quadratic change with a decrease and then an increase with increasing *stevia* straw addition (*p* = 0.03); the 15% and 25% groups were significantly lower than the CK group, with reductions of 11.55% and 5.33%, respectively (*p* < 0.05). Meat pH also followed a quadratic curve, but with an initial increase followed by a decrease with increasing *stevia* straw addition (*p* = 0.04); the 15% group was significantly higher than the CK and 35% groups, increasing by 0.99% and 0.83% (*p* < 0.05).

### 3.3. Conventional Chemical Characteristics of the Longissimus Dorsi Muscle in Lambs

As shown in Table 5, EE content of the longissimus dorsi muscle showed a linear increase with an increasing percentage of *stevia* straw (*p* < 0.01); the experimental groups were significantly or highly significantly higher than the CK group, with increases of 52.76%, 54.77%, 82.41%, and 107.53%, respectively (*p* < 0.05 or *p* < 0.01); the cholesterol content of the 25% and 35% groups was highly significantly higher than that of the CK group (*p* < 0.05), increasing by 59.40% and 63.28%, respectively.

### 3.4. Effect of Stevia Straw Supplementation on Amino Acid Content of Lamb Meat

As shown in Table 6, the glutamic acid content of the 15% group was significantly higher than that of the 25% group (*p* < 0.05), with an increase of 154.27%. The 5% group histidine content is extremely significantly higher than that of the 35% group (*p* < 0.05), increased by 56.59%.

### 3.5. Effect of Stevia Straw Supplementation on Fatty Acid Content of Lamb Meat

Table 7 shows that the C12:0 content followed a quadratic curve with increasing addition of *stevia* straw (*p* = 0.01) and the 35% group was significantly higher than the 5% and 15% groups (*p* < 0.05). The content of C16:0 linearly decreased with increased *stevia* straw addition up to 25% (*p* = 0.02) and the CK and 5% groups were significantly higher than the 25% group (*p* = 0.03). The C18:1n9c content showed a quadratic response (increase and then decrease) to increased *stevia* straw addition (*p* = 0.03), and the C18:1n9c content of the 5%, 15% and 25% groups was significantly higher than the CK group (*p* < 0.05). The content of C18:2n6c in the 25% group was significantly higher than that in the CK, the 5%, and the 15% groups.

### 3.6. Effect of Stevia Straw Supplementation on Rumen Microbial Diversity

#### 3.6.1. Analysis of OTUs in the Rumen Microbial Community

**Figure 2:**
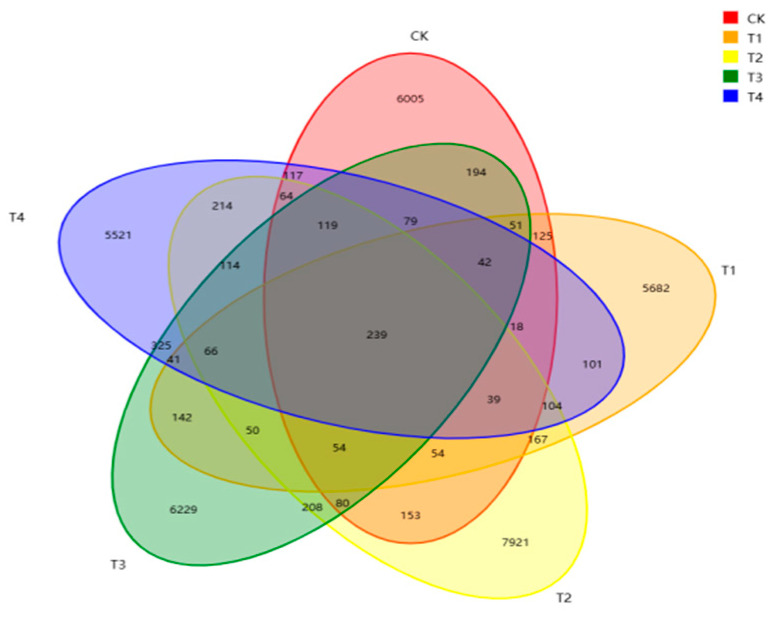
Venn diagram of rumen fluid microbiota in lambs supplemented with different percentages of *stevia* straw. CK = control group, T1 = 5% group, T2 = 15% group, T3 = 25% group, and T4 = 35% group.

The determination of OTUs indicated that the rumen fluid of fattening lambs had a total of 239 overlapping microbial OTUs in the CK, 5%, 15%, 25%, and 35% groups (Figure 2). Individually, a total of 6005 OTUs were detected in the CK group; 5682 OTUs in the 5% group; 7921 OTUs in the 15% group; 6229 OTUs in the 25% group; and 5521 OTUs in the 35% group.

#### 3.6.2. Alpha Diversity Analysis

Table 8 shows that the addition of different levels of *stevia* straw to the feed did not have a significant effect on alpha diversity in the rumen fluid of fattening lambs (*p* > 0.05). The coverage rate of each group was >0.99, which indicated that the data of each group accurately reflected the composition of the rumen microbial community.

#### 3.6.3. Species Composition

As shown in Table 9, the top ten species in relative abundance at the phylum level of sheep rumen fluid were Bacteroidota, Firmicutes, Proteobacteria, Halobacterota, Verrucomicrobiota, Actinobacteriota, Unclassified_ Bacteria, Patescibacteria, Spirochaetota, Crenarchaeota, and Others. The relative abundance of Verrucomicrobiota in the CK group was significantly higher than that in the 5% and 15% groups (*p* < 0.05). The 25% and 35% groups showed increased relative abundance of Bacteroidota and Firmicutes, but the differences were not significant (*p* > 0.05).

The top ten species in relative abundance at the family level of rumen fluid microbiota were Prevotellaceae, Lactobacillaceae, Lachnospiraceae, Comamonadaceae, Haloferacaceae, Acetobacteraceae, Rikenellaceae, Halomicrobiaceae, Muribaculaceae, Selenomonadaceae, and Others (Table 10). The relative abundance of Others in the CK group was significantly higher than that in the 5%, 15%, and 35% groups (*p* < 0.05).

As shown in Table 11, the top ten species in relative abundance at the genus level in rumen fluid were uncultured_rumen_bacterium, Prevotella, Paucibacter, Rikenellaceae_RC9_gut_group, Leuconostoc, Acetobacter, unclassified_Prevotellaceae, Unclassified_Bacteria, Succiniclasticum, Fructobacillus, and Others. Supplementation with different percentages of *stevia* straw did not significantly affect the abundance of bacteria at the genus level in the rumen fluid of lambs (*p* > 0.05).

#### 3.6.4. LEfSe Analysis

**Figure 3:**
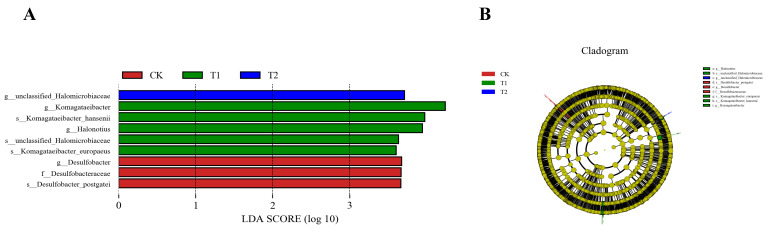
Histogram (**A**) and evolutionary branching diagram (**B**) of LDA value distribution for LEfSe analysis. CK: control group; T1: 5% group; T2: 15% group.

As shown in Figure 3, three species were significant in the CK group, Desulfobacter, Desulfobacteraceae, and Desulfobacter_postgatei; five species were significant in the 5% group, namely, Komagataeibacter, Komagataeibacter_hansenii, Halonotius, unclassified_Halomicrobiaceae, and Komagataeibacter_europaeus; and one species with significance in the 15% group was unclassified_Halomicrobiaceae.

## 4. Discussion

Slaughter performance is an important index for assessing the production of meat animals and poultry. It clearly reflects the proportion of edible meat in an adult sheep, and it is also a core criterion for measuring the production of lamb meat. It not only reflects the meat production capacity and organ development of livestock and poultry, but also indicates the digestion and utilization capacity of the ration by the animal [9]. Our investigation showed that with increasing *stevia* straw addition, the pre-slaughter live weight and carcass weight showed a quadratic curve change with an initial increase and then a decrease. The average carcass weight of each *stevia* straw group was higher than that of the CK group, but the difference was not significant, and the other indices were not significantly different. This indicates that the addition of *stevia* straw to the ration can improve the meat production performance of lambs, which may be related to the fact that *stevia* straw is relatively rich in chlorogenic acid. Chlorogenic acid is a natural bioactive substance that can enhance the bioavailability of dietary nutrients and promote the expression of transporter carriers, thus enhancing an animal’s ability to transport and absorb minerals, which could ultimately improve their slaughter performance [10,11].

After slaughter, the pH of lamb meat gradually decreases with increasing time and glycolytic reactions. This is due to the depletion of muscle glycogen for anaerobic respiration by muscle cells after slaughter, which produces excess lactic acid leading to a decrease in pH [12,13]. The cooking loss and water loss rates are important indicators of muscle water-holding capacity; the higher the cooking loss and the lower the water loss rate, the higher the water-holding capacity of the muscle and the better the meat quality [14]. The water-holding capacity of muscle is closely related to pH, and a lower pH may lead to changes in muscle structure that affect its water retention [15]. Therefore, a decrease in pH can indirectly reduce meat quality by increasing its water loss rate.

The results of our experiments showed that the water loss rate and cooking loss of the 15% and 25% *stevia* straw groups were significantly lower than the control group. The pH of the 15% group was significantly higher than that of the CK group and the 25% group, and it showed a quadratic response of increasing and then decreasing with the increased addition of *stevia* straw. This supports our hypothesis that the feeding of *stevia* straw improves the water retention capacity of lamb meat. This may be related to the presence of active substances such as flavonoids and alkaloids in *stevia* straw, which are able to slow down the rate of oxidation of muscle lipids and maintain the structural integrity of muscle cell membranes [16].

*Stevia* straw is rich in flavonoids and polyphenols, which not only have antioxidant effects, but can also effectively improve the nutrient composition of livestock muscle and enhance the meat’s nutritional value [17]. Duan et al. [18] added residual black wolfberry fruit to the diets of sheep and reported a significant increase in the content of crude fat in the muscle, which improved its nutritional value. Our study showed that the crude fat content of each experimental group was significantly higher than that of the CK group, and linearly increased with *stevia* straw addition. Based on the above, we hypothesize that the dietary inclusion of stevia straw reduced both the cooking loss percentage and water loss percentage in the mutton longissimus dorsi, which may be attributed to the significant increase in intramuscular fat content. The elevated fat content enhances the muscle’s water-holding capacity, as moisture in the muscle is partially physically replaced by fat, thereby reducing water loss during cooking and resulting in a lower cooking loss percentage [19,20].

In our experiment, the longissimus dorsi muscle of lambs was selected for fatty acid composition testing, and it was found that the addition of *stevia* straw to the ration increased the C12:0, C18:2n6c, C18:1n9c, and C20:1 content of the muscle, and decreased the C16:0 and C18:0 levels. This may be due to the fact that *stevia* straw is rich in chlorogenic acid and phenolics, which can regulate the activity of enzymes involved in fat metabolism by activating the AMPK signaling pathway, thus controlling the synthesis and metabolism of fatty acids in the organism [21]. This phenomenon confirms stevia as an anti-aging product [22]; indeed, increase in C:16 has been reported in the meat of older animals [23]. *Stevia* supplementation can also promote the activity of a desaturase located in the endoplasmic reticulum. By the generation of acetyl-coenzyme A from acetic acid, it can catalyze the conversion of stearic acid to oleic acid, thus reducing the saturated fatty acid content and increasing the unsaturated fatty acid content which improves lamb meat quality. When *stevia* straw was added at 25%, it significantly increased the content of unsaturated fatty acids and decreased the content of saturated fatty acids in the longissimus dorsi muscle. This is consistent with the results of Kwiecien et al. [24], who found that the bioactive components in alfalfa could increase the saturated fatty acid enzyme activity and promote the conversion of saturated fatty acids to polyunsaturated fatty acids. This may be the result of flavonoid activity that inhibits the synthesis of phospholipids and free fatty acids, which promotes an increase in unsaturated fatty acids and improved meat quality. Moreover, the results of this study showed that the addition of 15% *stevia* straw could significantly increase the glutamic acid content in the longissimus dorsi muscle of lambs. *Stevia* straw is rich in glycosides, which can significantly increase the content of flavor-promoting amino acids in the muscle and improve the taste of the lamb [25,26].

The rumen microbiota of ruminants comprise a highly complex ecosystem with multiple functions such as fiber catabolism, fat degradation, plant protein hydrolysis and microbial protein synthesis [27,28]. These microorganisms play a key role in nutrient absorption and distribution, metabolism, and the immune responses of the organism [28,29]. Our Venn diagram analysis based on the level of OTUs in this study found that the number of rumen-specific OTUs was higher in the 15% group than in the other groups. The level of rumen microbial diversity was also higher than that of the other groups, suggesting that feeding *stevia* straw can increase rumen microbial diversity. The rumen microbiota composition is an important part of the digestive system of ruminants, and the structure and type of diet is one of the key factors affecting rumen microecological balance. In this study, the relative abundances of Firmicutes and Bacteroidetes were the highest, which is consistent with the rumen fermentation results. Bacteroidetes primarily convert non-fibrous substances into acetic acid [30], propionic acid, and other compounds. Firmicutes can produce various digestive enzymes to break down nutrients and are key bacterial groups for improving fiber utilization [31]. We hypothesize that the high fiber content in stevia straw leads to changes in the rumen environment, which can increase the relative abundance of cellulose-degrading bacteria. These bacteria secrete large amounts of cellulase to break down the cellulose in the straw, thereby increasing the concentration of propionic acid and promoting energy supply.

## 5. Conclusions

Adding 15% to 25% *stevia* straw to the ration can significantly improve the slaughtering performance, meat quality and muscle nutrient composition of fattening sheep. In addition, the addition of *stevia* straw had no negative effect on the rumen microflora, and the 15% level could increase the abundance and activity of the rumen microflora, so it is recommended to use 15% *stevia* straw in the ration for sheep during the fattening period.

## Figures and Tables

**Table 1:** Nutritional composition of *stevia* straw.

Items	DM	CP	EE	NDF	ADF	Ash	Ca	P
Nutritional content	96.72	4.06	1.02	59.86	42.06	4.54	0.28	0.11

Note: Nutrient contents are measured as % of DM, expressed on a DM basis, which is measured on the basis of fresh weight. CP, crude protein; EE, ether extract; NDF, neutral detergent fiber; ADF, acid detergent fiber; Ca, calcium; P, phosphorus.

**Table 2:** Composition and nutrient levels of experimental diets (DM basis).

Items	Groups				
	Control Group	5% Group	15% Group	25% Group	35% Group
Ingredients					
Stevia straw	0.00	4.99	15.02	25.01	35.00
Alfalfa hay	14.21	12.9	9.79	5.53	2.05
Wheat stalk	24.38	20.84	14.07	8.32	1.84
Corn	32.54	32.37	32.23	32.21	32.15
Wheat bran	6.93	6.86	6.86	6.84	6.83
Cottonseed meal	14.43	14.52	14.53	14.57	14.66
NaCl	0.96	0.96	0.95	0.96	0.94
NaHCO_3_	1.55	1.56	1.55	1.56	1.53
Premix	5.00	5.00	5.00	5.00	5.00
Total	100.00	100.00	100.00	100.00	100.00
Nutritional level					
ME/(MJ/kg)	10.49	10.55	10.35	10.50	10.43
CP (%)	13.53	13.59	13.66	13.54	13.46
NDF (%)	33.09	32.63	32.23	33.01	33.63
ADF (%)	20.42	20.04	18.82	20.42	21.06
EE (%)	2.31	2.32	2.68	2.17	2.59
Ca (%)	0.82	0.82	0.86	0.82	0.80
P (%)	0.40	0.41	0.51	0.43	0.43

Notes: (1) The premix provides 10,000 IU of vitamin A, 1000 IU of vitamin D, 200 IU of vitamin E, 145 mg of iron, 80 mg of zinc, 20 mg of copper, 98 mg of manganese, 2.5 mg of iodine, 0.35 mg of selenium, 0.65 mg of cobalt. (2) Metabolic energy is the calculated value, and the other indicators are measured values. ME (MJ/kg DM) = 0.0460.820 × DE (MJ/kg DM).

**Table 3:** Effect of different dietary levels of *stevia* straw on the slaughter performance of lambs.

Item	Groups					SEM	*p*-Value		
	CK	5%	15%	25%	35%		Total	Linear	Quadratic
Live weight before slaughter (kg)	35.52 ^b^	40.25 ^ab^	44.35 ^a^	41.17 ^a^	40.85 ^ab^	0.960	0.028	0.048	0.016
Carcass weight (kg)	18.00	20.90	23.23	21.28	20.00	0.596	0.051	0.087	0.022
Carcass dressing (%)	50.72	51.85	52.58	51.57	51.43	0.004	0.807	0.744	0.863
Head and hoof weight (kg)	3.23	3.49	3.62	3.72	3.55	0.055	0.151	0.050	0.090
Loin eye area (cm^2^)	18.24	19.68	20.53	18.72	18.09	0.726	0.839	0.823	0.315
Back fat thickness (mm)	4.09	4.93	4.12	4.01	4.23	0.356	0.941	0.817	0.868

^a, b^ Different superscripts indicate significant differences within a row (*p* < 0.05).

**Table 4:** Effect of different levels of *stevia* straw on the quality of lamb meat.

Items	Groups					SEM	*p*-Value		
	CK	5%	15%	25%	35%		Total	Linear	Quadratic
Water loss (%)	41.14 ^a^	39.49 ^abc^	38.29 ^c^	39.19 ^bc^	40.73 ^ab^	0.313	0.012	0.547	0.001
Cooking loss (%)	30.21 ^a^	29.20 ^a^	26.72 ^b^	28.60 ^b^	29.73 ^a^	0.386	0.025	0.502	0.005
pH	6.03 ^c^	6.06 ^abc^	6.09 ^a^	6.07 ^ab^	6.04 ^bc^	0.006	0.012	0.476	0.001

^a, b, c^ Different superscripts indicate significant differences within a row (*p* < 0.05).

**Table 5:** Effect of dietary *stevia* straw supplementation on chemical characteristics of the longissimus dorsi muscle of lambs.

Item	Groups					SEM	*p*-Value		
	CK	5%	15%	25%	35%		Total	Linear	Quadratic
Moisture (%)	76.44	74.95	75.92	74.02	74.19	0.479	0.444	0.125	0.914
EE (%)	1.99 ^Bc^	3.04 ^ABb^	3.08 ^ABb^	3.63 ^Aab^	4.13 ^Aa^	0.203	0.001	0.001	0.459
CP (%)	19.69	20.25	20.67	20.09	19.52	0.375	0.906	0.805	0.971
Ash (%)	4.68	4.37	4.64	4.61	4.44	0.095	0.836	0.733	0.963
Cholesterol (mg/100 g)	332.75 ^Bb^	440.16 ^ABab^	449.70 ^ABab^	530.42 ^Aa^	543.31 ^Aa^	22.432	0.009	0.001	0.439
Thiamine (mg/kg)	1.08	0.83	0.88	0.89	1.03	0.041	0.649	0.296	0.304
Inosinic acid (mg/kg)	401.92	425.44	563.80	512.22	462.11	38.420	0.810	0.926	0.272

^A, B^ Different superscripts indicate significant differences within a row (*p* < 0.01); ^a, b, c^ different superscripts indicate significant differences within a row (*p* < 0.05).

**Table 6:** Effect of different percentages of dietary *stevia* straw supplementation on amino acid content in the longissimus dorsi muscle of lambs (μg/g).

Items	Groups					SEM	*p*-Value		
	CK	5%	15%	25%	35%		Total	Linear	Quadratic
Gly	216.40	191.95	233.17	217.21	191.02	7.835	0.390	0.648	0.362
Ala	550.20	564.62	579.16	566.40	571.91	18.184	0.993	0.750	0.788
Ser	75.73	67.94	85.33	73.82	71.10	2.759	0.353	0.862	0.419
Pro	40.69	38.14	44.77	40.06	37.25	1.222	0.351	0.567	0.253
Val	43.17	38.80	39.14	41.46	35.74	1.222	0.390	0.170	0.944
Thr	59.28	49.84	53.23	54.38	43.69	2.101	0.192	0.073	0.778
Ile	29.03	28.91	28.93	29.59	26.89	0.659	0.774	0.471	0.445
Leu	56.68	58.24	53.88	58.29	54.24	1.409	0.801	0.650	0.846
Asn	58.76	41.38	54.82	49.34	44.10	2.609	0.185	0.235	0.798
Orn	10.66	6.70	16.26	10.99	14.63	1.639	0.409	0.302	0.978
Gln	707.27	418.87	736.23	561.25	674.67	61.115	0.486	0.861	0.553
Lys	61.27	61.32	68.13	68.14	58.79	2.238	0.595	0.909	0.198
Glu	26.39 ^b^	27.90 ^b^	52.71 ^a^	20.73 ^b^	28.01 ^ab^	4.265	0.021	0.890	0.187
Met	22.57	26.10	24.84	27.01	23.69	0.936	0.606	0.649	0.216
His	233.48 ^ab^	317.59 ^a^	261.16 ^ab^	276.54 ^ab^	202.82 ^b^	13.778	0.045	0.254	0.028
Phe	39.30	43.54	40.12	45.08	40.25	1.208	0.516	0.695	0.351
Arg	92.42	75.97	93.63	89.71	84.73	3.727	0.596	0.953	0.967
Tyr	36.85	38.57	39.42	39.82	36.33	1.191	0.876	0.982	0.318
Trp	7.33	7.99	8.20	8.19	8.03	0.193	0.644	0.270	0.285
SAA	1003.56	973.81	1063.79	1020.01	973.77	27.039	0.847	0.948	0.495
DAA	822.01	813.38	893.96	833.94	817.83	24.067	0.849	0.947	0.475
TAA	2367.48	2104.38	2513.11	2278.02	2247.88	81.203	0.631	0.913	0.475
EAA	311.29	306.75	308.26	323.95	283.28	7.994	0.641	0.513	0.410
NEAA	1272.16	1322.69	1389.35	1324.31	1223.17	32.094	0.588	0.683	0.129

^a, b^ Different superscripts indicate significant differences within a row (*p* < 0.05).

**Table 7:** Effect of different percentages of dietary *stevia* straw supplementation on fatty acid content in the longissimus dorsi muscle of lambs (%).

Items	Groups					SEM	*p*-Value		
	CK	5%	15%	25%	35%		Total	Linear	Quadratic
C10:0	0.19	0.18	0.2	0.2	0.21	0.003	0.294	0.067	0.686
C12:0	0.15 ^ab^	0.12 ^b^	0.13 ^b^	0.15 ^ab^	0.22 ^a^	0.011	0.019	0.015	0.012
C14:0	3.03	2.49	2.64	2.66	3.37	0.108	0.056	0.220	0.008
C14:1	0.39	0.27	0.34	0.49	0.42	0.008	0.401	0.280	0.330
C15:0	0.39	0.27	0.34	0.49	0.42	0.025	0.062	0.107	0.325
C16:0	28.85 ^a^	27.88 ^a^	27.18 ^ab^	25.66 ^b^	27.46 ^ab^	0.321	0.016	0.014	0.044
C16:1	2.01	1.79	1.72	2.29	2.04	0.083	0.210	0.335	0.397
C17:0	1.30	1.06	1.29	1.58	1.28	0.058	0.078	0.208	0.875
C18:0	20.42	19.01	19.84	16.96	18.06	0.555	0.301	0.093	0.772
C18:1n9c	39.83 ^b^	43.60 ^a^	43.02 ^a^	43.82 ^a^	42.87 ^ab^	0.471	0.034	0.039	0.026
C18:2n6c	3.17 ^b^	3.00 ^b^	2.96 ^b^	4.85 ^a^	3.41 ^ab^	0.232	0.040	0.117	0.716
C20:0	0.10	0.09	0.09	0.07	0.09	0.006	0.647	0.558	0.772
C20:1	0.01 ^Bb^	0.06 ^Aa^	0.05 ^Aa^	0.06 ^Aa^	0.07 ^Aa^	0.006	0.007	0.002	0.156
C18:3n3	0.27	0.23	0.32	0.83	0.15	0.089	0.105	0.539	0.212
C20:4n6	0.20 ^ab^	0.15 ^b^	0.15 ^b^	0.23 ^a^	0.24 ^a^	0.011	0.011	0.033	0.014
SFA	54.42 ^a^	51.09 ^ab^	51.70 ^ab^	47.77 ^b^	51.10 ^ab^	0.702	0.041	0.031	0.099
UFA	45.58 ^b^	48.92 ^ab^	48.30 ^ab^	52.21 ^a^	48.90 ^ab^	0.701	0.042	0.031	0.100
UFA/SFA	0.84	0.96	0.94	1.09	0.96	0.030	0.051	0.049	0.120

^A, B^ Different superscripts indicate significant differences within a row (*p* < 0.01); ^a, b^ different superscripts indicate significant differences within a row (*p* < 0.05).

**Table 8:** Effect of different levels of *stevia* straw on rumen microbial alpha diversity in lambs.

Items	Groups					SEM	*p*-Value		
	CK	5%	15%	25%	35%		Total	Linear	Quadratic
Feature	1589.60	1449.40	2061.40	1775.00	1542.00	160.478	0.798	0.849	0.452
ACE	1599.11	1458.14	2073.17	1788.39	1555.25	161.018	0.798	0.842	0.454
Chao1	1590.36	1450.48	2062.3	1776.42	1543.41	160.448	0.798	0.848	0.454
Simpson	0.98	0.94	0.99	0.98	0.98	0.008	0.266	0.639	0.739
Shannon	7.89	6.52	8.61	8.15	7.79	0.328	0.357	0.541	0.849
PD whole tree	310.26	370.82	344.02	434.24	314.65	38.088	0.865	0.803	0.481
Goods coverage	0.99	0.99	0.99	0.99	0.99	0.000	0.500	0.102	0.547

**Table 9:** Effects of different levels of *stevia* straw on the abundance of microbiota at the rumen phylum level in lambs.

Items	Groups					SEM	*p*-Value		
	CK	5%	15%	25%	35%		Total	Linear	Quadratic
Bacteroidota	27.51	12.19	24.01	31.77	33.43	3.283	0.265	0.177	0.273
Firmicutes	19.38	30.73	27.22	32.78	28.96	12.356	0.513	0.245	0.322
Proteobacteria	30.07	29.51	11.42	14.96	12.17	4.028	0.383	0.090	0.613
Halobacterota	0.68	13.22	18.44	1.81	9.22	3.438	0.454	0.818	0.279
Verrucomicrobiota	4.75 ^a^	1.06 ^b^	2.07 ^b^	2.71 ^ab^	2.91 ^ab^	0.397	0.037	0.413	0.017
Actinobacteriota	3.54	3.61	4.53	3.29	4.16	3.283	0.265	0.177	0.273
Patescibacteria	1.07	0.52	1.34	1.93	0.48	0.103	0.091	0.857	0.186
Spirochaetota	0.74	0.46	0.98	1.40	0.66	0.289	0.403	0.484	0.444
Crenarchaeota	0.09	1.90	2.52	0.82	2.54	0.702	0.782	0.472	0.691
Others	8.99	4.36	4.68	6.08	3.17	0.908	0.262	0.108	0.524

^a, b^ Different superscripts indicate significant differences within a row (*p* < 0.05).

**Table 10:** Effect of different levels of *stevia* straw on the abundance of rumen microbiota at the family level in lambs.

Items	Groups					SEM	*p*-Value		
	CK	5%	15%	25%	35%		Total	Linear	Quadratic
Prevotellaceae	10.12	9.88	6.45	12.05	11.87	2.123	0.268	0.083	0.400
Lactobacillaceae	0.49	21.51	3.75	5.11	4.61	2.706	0.100	0.643	0.259
Lachnospiraceae	5.26	2.34	4.81	7.18	5.44	0.835	0.513	0.395	0.755
Comamonadaceae	1.21	10.22	3.22	5.09	4.85	1.547	0.469	0.846	0.468
Haloferacaceae	0.44	6.12	8.17	1.22	5.28	1.680	0.577	0.699	0.405
Acetobacteraceae	0.93	13.11	2.08	2.43	2.50	1.663	0.110	0.492	0.326
Rikenellaceae	4.40	1.69	3.78	4.27	6.02	0.761	0.533	0.299	0.219
Halomicrobiaceae	0.19	6.50	8.83	0.56	3.54	1.632	0.400	0.949	0.219
Muribaculaceae	1.57	1.57	4.07	1.56	3.39	0.479	0.287	0.273	0.728
Selenomonadaceae	1.39	0.56	2.27	4.49	3.27	0.535	0.146	0.041	0.949
Others	72.85 ^a^	31.00 ^b^	48.42 ^ab^	50.15 ^ab^	42.84 ^b^	4.513	0.040	0.153	0.116

^a, b^ Different superscripts indicate significant differences within a row (*p* < 0.05).

**Table 11:** Effect of different levels of *stevia* straw on the abundance of microbiota at the genus level in lambs.

Items	Groups					SEM	*p*-Value		
	CK	5%	15%	25%	35%		Total	Linear	Quadratic
*uncultured_rumen_bacterium*	5.55	2.21	4.08	8.03	7.82	1.092	0.411	0.193	0.372
*Prevotella*	5.42	3.03	3.96	8.49	7.81	1.031	0.399	0.173	0.423
*Paucibacter*	0.54	9.69	3.67	4.77	4.55	4.322	0.497	0.783	0.388
*Rikenellaceae_RC9_gut_group*	4.04	1.48	2.54	4.09	5.86	0.721	0.390	0.232	0.143
*Leuconostoc*	0.00	8.86	1.52	2.95	2.59	1.247	0.211	0.931	0.343
*Acetobacter*	0.02	8.85	1.69	1.99	2.09	1.233	0.186	0.744	0.316
*unclassified_Prevotellaceae*	2.05	0.61	2.04	1.77	6.52	0.907	0.297	0.122	0.164
*Succiniclasticum*	1.17	1.36	3.69	2.78	2.38	0.505	0.523	0.300	0.314
*Fructobacillus*	0.00	6.57	1.04	1.42	1.36	0.833	0.089	0.651	0.265
*Others*	78.02	54.89	71.04	61.26	56.73	4.041	0.327	0.211	0.737

## Data Availability

The original contributions presented in this study are included in the article. Further inquiries can be directed to the corresponding author.
